# Long Non-Coding RNA CCAT2 Activates RAB14 and Acts as an Oncogene in Colorectal Cancer

**DOI:** 10.3389/fonc.2021.751903

**Published:** 2021-11-19

**Authors:** Dalu Wang, Zhilong Li, Hongzhuan Yin

**Affiliations:** Department of General Surgery, Shengjing Hospital of China Medical University, Shenyang, China

**Keywords:** LncRNA, CCAT2, colorectal cancer, TAF15, RAB14

## Abstract

Here, we investigated the clinicopathological and prognostic potential of the long noncoding RNA Colon Cancer-Associated Transcript 2 (CCAT2) in human colorectal cancer (CRC). We used qPCR to quantify CCAT2 levels in 44 pairs of CRC tissues and adjacent nontumor and healthy colon mucosa tissues, and in several CRC cell lines (SW620, SW480, HT-29, LOVO, HCT116 and DLD-1) and normal human colorectal epithelial cells (HFC). We assessed the effects of CCAT2 overexpression or knockdown on the proliferation, migration and invasion by SW620 and LOVO cells using CCK-8, transwell, and wound−healing assays, respectively. We also investigated the potential interaction between CCAT2 and TAF15 through RNA pull down and rescue experiments. Lastly, we evaluated the expression of the cell cycle progression markers and GSK3β signaling pathway proteins using Western blotting. Our results showed that CCAT2 was upregulated in CRC tissues and cell lines as com-pared to controls. Ectopic expression of CCAT2 promoted CRC cell proliferation, migration and invasion, likely through direct interaction with TAF15, transcriptional activation of RAB14, and activation of the AKT/GSK3β signaling pathway. *In vivo*, CCAT2 promoted CRC cell growth and metastasis in nude mice. Taken together, these results highlight the actions of CCAT2 as a CRC oncogene.

## Introduction

Colorectal cancer (CRC) is the third most common malignancy worldwide affecting more than 1.2 million people every year and is the fourth leading cause of cancer-associated mortality, causing more than 600,000 yearly deaths (1). CRC incidence is higher among men than among women and strongly increases with age (2). CRC has both hereditary and environmental causes that contribute to the gradual development of the disease through the adenoma-carcinoma sequence. CRC therapies include surgery, adjuvant radiotherapy, adjuvant chemotherapy, fluorouracil-based chemotherapy, and oxaliplatin adjuvant treatment, among others, which are applied depending on the pathological stage of each patient (3, 4). Some patients develop radioresistance resulting in poor prognosis, which could be alleviated by early detection (5).

Recently, non-coding RNAs have been proposed as potential diagnostic and prognostic biomarkers for several types of cancer (6, 7). Long non-coding RNAs (lncRNAs) can function as decoy, scaffold, guide, and enhancer RNAs, and participate as short nucleic acid strands in chromatin remodeling, transcriptional and post-transcriptional regulation, and epigenetics (8–10). Several lncRNAs have been linked to various types of cancer; for example, terminal differentiation-induced non-coding RNA (TINCR), lncRNA-p21, lncRNA OIP5-AS1, lncRNA UCA1 and Hox transcript antisense intergenic RNA (HOTAIR) have been identified as potential therapeutic targets for cancer treatment (11, 12). In addition, lncRNA such as RC3H2, TANRIC, PTENP1 FOXD2-AS1 has been identified as diagnostic or prognostic predictor for various types of cancer (13–16).

LncRNA colon cancer-associated transcript-2 (CCAT2) expression is cell- and tissue-specific and localizes mainly to the nucleus of cells. CCAT2 is a 1752-base RNA transcribed from the 8q24 region of the human genome containing a single nucleotide polymorphism (SNP), rs6983267. The rs6983267 SNP has been associated with an increased risk of colorectal, prostate, ovarian and breast cancers (17–19). The genomic region spanning rs6983267 contains DNA enhancer elements that bind to transcription factor 7-like 2 (TCF7L2) and β-Catenin, and induce the production of cancer stem cell (CSCs). Overexpression of CCAT2 has been linked to various types of cancer, including CRC, breast, lung, esophageal squamous cell carcinoma, and gastric cancers. Indeed, this lncRNA promotes tumor growth and metastasis while causing reduced sensitivity to chemotherapy (20–26).

In this study, we investigated lncRNA CCAT2 expression in CRC tissues and cell lines. *In vitro*, CCAT2 overexpression promoted cell proliferation, migration and invasion by activating the RAB14 transcription factor and the AKT/GSK3β signaling pathway in CRC tissues and cell lines. Our results suggest that elements in the CCAT2/RAB14/AKT/GSK3β axis may serve as potential prognostic and diagnostic biomarkers to treat CRC.

## Results

### CCAT2 Expression Was Upregulated in CRC and Correlated With Lymph Node Metastasis

**Figure 1:**
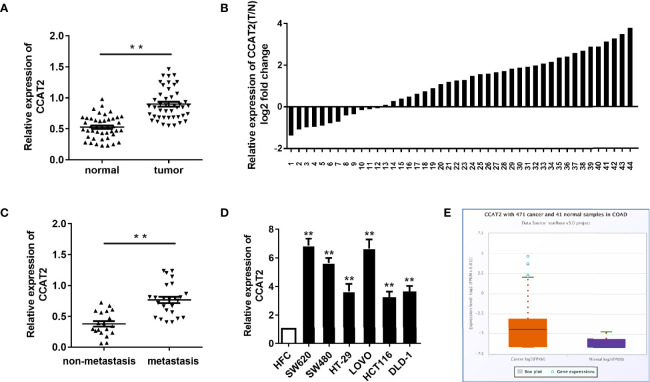
CCAT2 expression was upregulated in CRC and associated with lymph node metastasis. **(A, B)** CCAT2 levels in CRC tissues and corresponding normal tissues from 44 patients were examined by qPCR. Data are shown as log2 of fold change in B. **(C)** The xpression of CCAT2 in CRC with lymph node metastasis or not was detected by qPCR. **(D)** The expression of CCAT2 was upregulated in the CRC cell lines (SW620, SW480, HT-29, LOVO, HCT116 and DLD-1) as compared to the normal colorectal epithelial cell line (HFC). **(E)** Expression of CCAT2 in CRC according to the TCGA Database. Boxplots were based on 471 colorectal cancer samples and 41 normal samples. The data are represented as mean ± SD. Three independent biological repeats were used for each analysis. *P < 0.05, **P < 0.01 *vs* HFC.

In order to assess CCAT2 expression, we first examined the levels of CCAT2 in 44 paired colorectal cancer (CRC) and adjacent normal tissues *via* qPCR. Our results revealed that CCAT2 was upregulated in CRC tissues (**Figures 1A, B**). Furthermore, we measured CCAT2 levels in CRC with and without lymph node metastasis and found that CCAT2 was upregulated in the former case but not the latter (**Figure 1C**). In addition, qPCR also showed upregulation of CCAT2 in a CRC cell line (**Figure 1D**). The Cancer Genome Atlas (TCGA) database also indicated elevated CCAT2 expression in CRC (**Figure 1E**). Together, these results demonstrated that CCAT2 was upregulated in CRC and promoted metastasis, suggesting that CCAT2 might act as an oncogene in CRC.

### Ectopic Expression of CCAT2 Promoted Cell Proliferation in CRC

**Figure 2:**
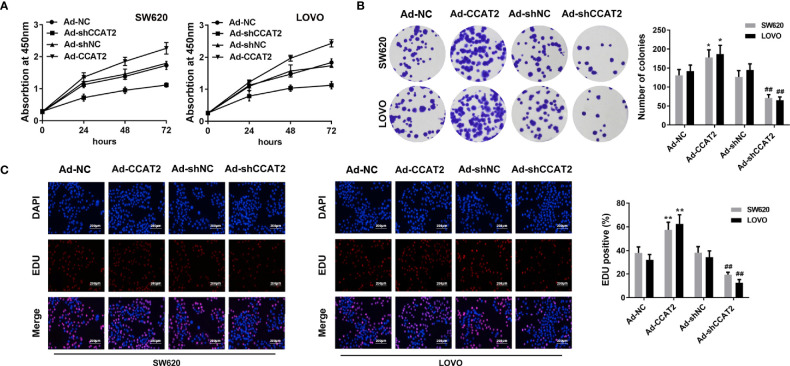
Overexpression of CCAT2 promoted cell proliferation in CRC. The SW620 and LOVO cells were infected with Ad-CCAT2, Ad-shCCAT2 or empty vector. **(A)** Cell viability was assessed through CCK8 assay. **(B)** Colony formation ability was enhanced in SW620 and LOVO cells overexpressing CCAT2 while being reduced by CCAT2 knockdown, as shown by cell colony assay. Data are represented as mean ± SD. **(C)** Cell proliferation was evaluated by EdU staining (×400). The data are represented as mean ± SD. Three independent biological repeats were used for each analysis. *P < 0.05, **P < 0.01 *vs* Ad-NC, ^##^P < 0.01 *vs* Ad-shNC.

CCK8 and colony-forming assay showed that the ectopic expression of CCAT2 enhanced the proliferation and colony formation of SW620 and LOVO cells (**Figures 2A, B**). However, the knockdown of CCAT2 had the reverse effects. In addition, we performed EdU staining to further validate these findings. Expectedly, our results showed a higher presence of EdU-positive cells among those with elevated expression of CCAT2, and knockdown of CCAT2 dramatically reduced the number of EdU-positive cells (**Figure 2C**). Thus, these results indicated that CCAT2 promoted colorectal cancer cell proliferation *in vitro*.

### Ectopic Expression of CCAT2 Promoted Colorectal Cancer Cell Invasion, Migration and Wound-Healing

**Figure 3:**
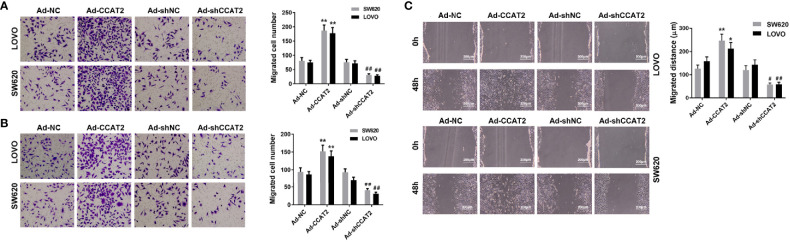
Ectopic expression of CCAT2 promoted colorectal cancer cell migration and invasion. Transwell assay was performed to assess the migration **(A)** and invasion **(B)** of CRC cells. CCAT2 promotes cell migration and invasion in SW620 and LOVO cells (×400). **(C)** Cell migration was further evaluated using wound healing assay. CCAT2 accelerate the healing of SW620 and LOVO cells (×400). The data are represented as mean ± SD. Three independent biological repeats were used for each analysis. *P < 0.05, **P < 0.01 *vs* Ad-NC, ^#^P < 0.05, ^##^P < 0.01 *vs* Ad-shNC.

To study whether CCAT2 is involved in colorectal cancer metastasis, we evaluated the effects of CCAT2 on the migration and invasion of colorectal cancer cells. Overexpression of CCAT2 promoted the migration and invasion of SW620 and LOVO cells (**Figures 3A, B**). Wound-healing assay revealed that colorectal cancer cells infected with Ad-CCAT2 migrated faster than those infected with Ad-NC while cells infected with Ad-sh-CCAT2 migrated more slowly than those infected with Ad-shNC (**Figure 3C**). The overexpression of CCAT2 promoted colorectal cancer cell invasion, migration and wound-healing.

### CCAT2 Promoted RAB14 Transcription Through Interaction With TAF15

**Figure 4:**
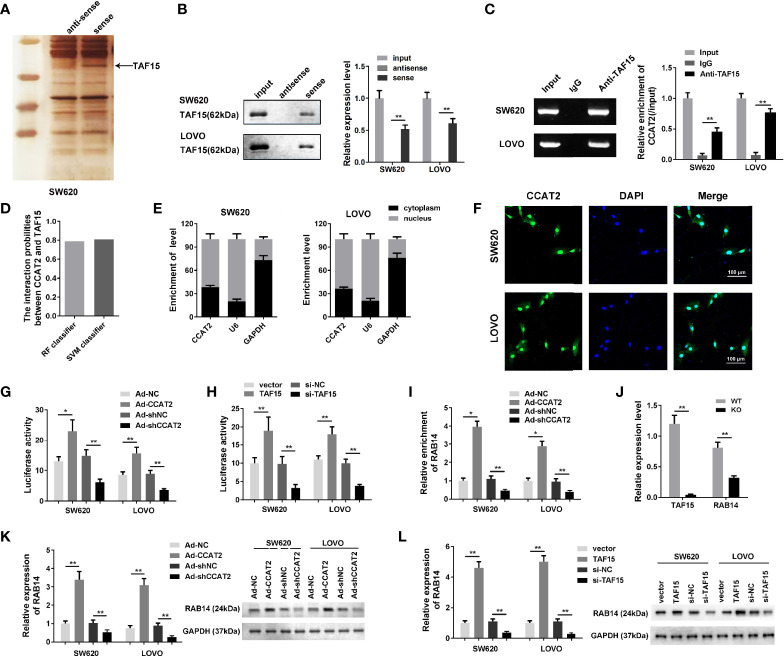
CCAT2 regulated the expression level of RAB14 *via* interaction with TAF15. **(A, B)** The RNA pull down assay was carried out by biotinylated CCAT2 probe and silver staining to reveal the factors underlying the mechanism of CCAT2 in CRC cells. Mass spectrometry analysis highlighted TAF15 as a potential factor interacting with CCAT2 **(A)**. The interaction of CCAT2 and TAF15 was further verified the by Western blot **(B)** and RIP assay **(C)**. **(D)** Bioinformatic prediction showing the potential interaction of CCAT2 and TAF15. **(E)** The cellular localization of CCAT2 as determined though nuclear and cytoplasmic separation test and qPCR. **(F)** FISH was performed to determine the localization of CCAT2 in the cells. **(G, H)** The transcriptional activity of RAB14 was measured by dual luciferase reporter assay in SW620 and LOVO cells after being infected or transfected with Ad-CCAT2, Ad-shCCAT2, TAF15, si-TAF15 and their corresponding controls. **(I)** ChIP analysis indicated that CCAT2 promoted the binding between ATF15 and the promtor of RAB14 while CCAT2 knockdown inhibited that. **(J)** Measurement of TAF15 and RAB14 levels by qPCR. Total RNA was extracted from SW620 cells that were transfected with CRISPR/Cas9 to knockdown TAF15. **(K, L)** Expression of RAB14 in cells infected with Ad-CCAT2, Ad-shCCAT2, TAF15, or si-TAF15 as measured by Western blot. Protein levels are shown as bar graphs. The data are represented as mean ± SD. Three independent biological repeats were used for each analysis. *P < 0.05, **P < 0.01.

To elucidate the molecular mechanism underlying the effects of CCAT2 on CRC cells, we performed RNA pull down and mass spectrometry analyses. We found that CCAT2 might directly bind with TAF15 (**Figures 4A, B**). Then, RIP assay was performed to further assess whether TAF15 could bind CCAT2 (**Figure 4C**). Furthermore, catRAPID software (http://s.tartaglialab.com/page/largeRNAs_group) was used to predict the potential interaction between CCAT2 and TAF15. Our results indicated a high probability of interaction between them (**Figure 4D**). We then performed nuclear and cytoplasmic isolation of SW620 and LOVO cells, and qPCR analysis revealed that CCAT2 was mainly located in the nucleus (**Figure 4E**), in agreement with the results of FISH experiments (**Figure 4F**). Therefore, we focused our study on the transcriptional regulation of CCAT2 and TAF15. Starbase software (http://starbase.sysu.edu.cn/index.php) highlighted RAB14 as a potential gene target of TAF15. To test whether TAF15 might transcriptionally activate RAB14, we used a luciferase reporter assay in SW620 and LOVO cells. Our results showed that enforced expression of CCAT2 and TAF15 dramatically increased the luciferase activity of the reporter vector containing the promoter region of RAB14 while knockdown of CCAT2 or TAF15 reduced that activity (**Figures 4G, H**). We then performed ChIP assay and the results indicated that CCAT2 overexpression promoted the binding between TAF15 and promoter of RAB14. On the contrast, CCAT2 silencing inhibited that (**Figure 4I**). Afterwards, SW620 cells were transfected with CRISPR-Cas9 to knock out TAF15 and total RNA extraction showed that the mRNA expressions of TAF15 and RAB14 were downregulated compared to those in wild type cells (**Figure 4J**). Furthermore, our results from Western blots showed that overexpression of CCAT2 or TAF15 increased the protein levels of RAB14 while knockdown of CCAT2 or TAF15 reduce the protein levels of RAB14 (**Figures 4K, L**). Taken together, these results suggest that CCAT2 interacts with TAF15 and promotes the expression of RAB14.

### Reduced Expression of RAB14 Inhibits the Proliferation-Enhancing Effect of CCAT2 on CRC Cells *In Vitro*

**Figure 5:**
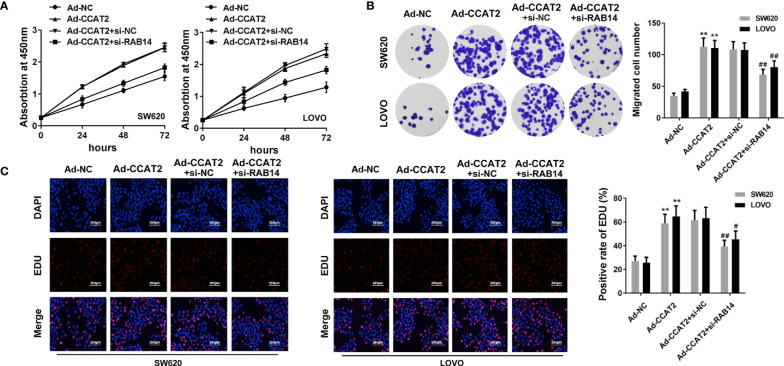
RAB14 knockdown inhibited the proliferation-enhancing effect of CCAT2 on CRC cells *in vitro*. **(A)** Cell viability was assessed by CCK8 assay. **(B)** RAB14 downregulation reversed the effect of CCAT2 on the colony formation ability of SW620 and LOVO cells. **(C)** Cell proliferation was evaluated by EdU staining (×400). The data are represented as mean ± SD. Three independent biological repeats were used for each analysis. **P < 0.01 *vs* Ad-NC, ^#^P < 0.05, ^##^P < 0.01 *vs* Ad-CCAT2+si-nc.

We transfected Ad-CCAT2-infected SW620 and LOVO cells with si-RAB14 to investigate effects of RAB14 on CCAT2 in CRC cells. After transfection, CCK8, colony formation, and EdU staining assays were employed to examine the proliferation of SW620 and LOVO cells. CCAT2 increased the proliferation of SW620 and LOVO cells, an effect that was reduced after transfection with si-RAB14, as strikingly apparent 72 h post-transfection (**Figure 5A**). Moreover, the colony (**Figure 5B**) and EdU-positive cell (**Figure 5C**) number were decreased after si-RAB14 transfection (**Figures 5B, C**). Taken together, our results suggested that RAB14 knockdown inhibited the proliferative effect of CCAT2 on CRC cells.

### RAB14 Knockdown Inhibits the Migration- and Invasion-Enhancing Effects of CCAT2 on CRC Cells

**Figure 6:**
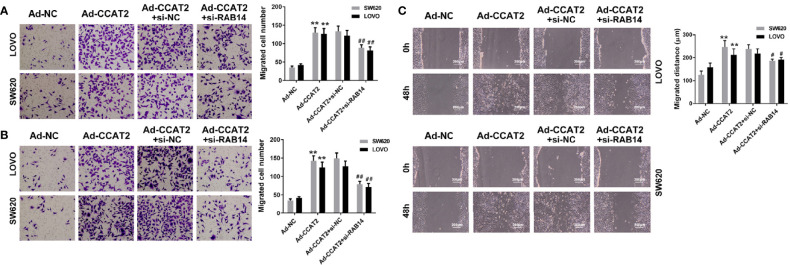
RAB14 knockdown inhibited the migration- and invasion-enhancing effects of CCAT2 on colorectal cancer cells *in vitro*. **(A, B)** Transwell (cell invasion and migration) assays and data quantification (×400). **(C)** Wound healing assays and data quantification (×400). The data are represented as mean ± SD. Three independent biological repeats were used for each analysis. **P < 0.01 *vs* Ad-NC, ^#^P < 0.05, ^##^P < 0.01 *vs* Ad-CCAT2+si-nc.

We further investigated the effects of CCAT2 on the migration and invasion of Ad-CCAT2-infected SW620 and LOVO cells transfected with si-RAB14. Our results showed that RAB14 knockdown inhibited the migration and invasion-enhancing effects of CCAT2 on CRC cells (**Figures 6A, B**). Furthermore, results from our wound-healing assays indicated that CRC cells co-transfected with si-RAB14 and Ad-CCAT2 displayed a reduced healing ability compared with those transfected with Ad-CCAT2 alone (**Figure 6C**).

### CCAT2 Activated the AKT/GSK3β Signaling Network, and Promoted Cell Cycle Progression and EMT *via* RAB14

**Figure 7:**
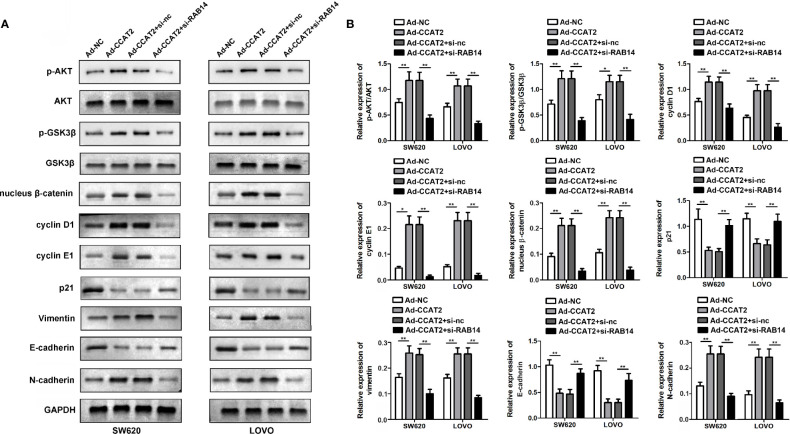
CCAT2 activated the AKT/GSK3β signaling pathway, promoted cell cycle and EMT, while these effects were rescued by RAB14. **(A, B)** Phosphorylation levels of AKT/GSK3β in response to ectopic CCAT2 in SW620 and LOVO cells. The Relative fold change in phosphorylation was calculated by the ratio of the phosphorylation signal over total protein signal of AKT and GSK3β. Simultaneously, the expressions of cell cycle and EMT-related factors were also quantified. The endogenous levels of GAPDH protein were used for normalization. The data are represented as mean ± SD. Three independent biological repeats were used for each analysis. *P < 0.05, **P < 0.01.

To better understand the mechanistic link among the factors underlying the regulation of CCAT2 in CRC, we tested whether the upregulation of CCAT2 affected the AKT/GSK3β signaling pathway. Our results indicated that CCAT2 overexpression enhanced AKT and GSK3β activity in SW620 and LOVO cells. We also measured the levels of Cyclin D1, Cyclin E1 and p21, which contribute to cell cycle progression and are important components of the Wnt/β−catenin signaling pathway. We found that CCAT2 activated the Wnt/β-catenin signaling pathway through activation of nuclear β-catenin. To further understand the underlying mechanism by which CCAT2 induced migration and invasion of SW620 and LOVO cells, we evaluated the expression of EMT-related proteins. CCAT2 upregulated the expression of Vimentin, and N-cadherin (**Figures 7A, B**). Moreover, these effects were rescued by RAB14. In brief, all these data indicated that CCAT2 might promote proliferation, migration and invasion by activating the RAB14/AKT/GSK3β signaling pathway in CRC cells.

### CCAT2 Promotes CRC Cell Growth and Metastasis in Nude Mice

**Figure 8:**
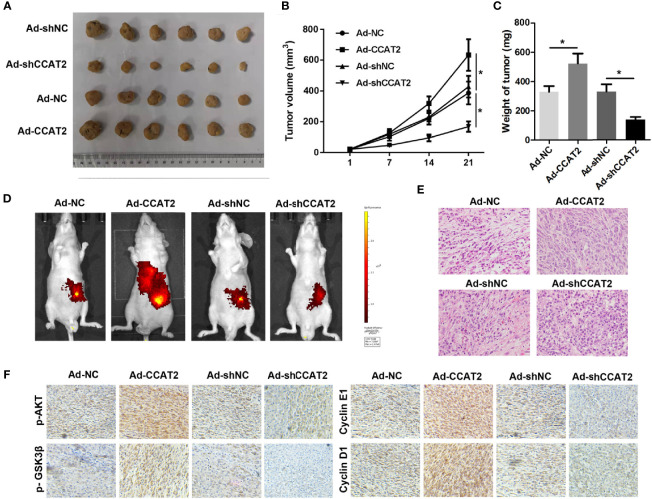
CCAT2 promoted the subcutaneous xenograft growth of colorectal cancer cells and hepatic metastasis in nude mice. **(A)** SW620 cells infected with Ad-CCAT2, Ad-shCCAT2 or their controls were subcutaneously injected into the back of nude mice. The tumors that developed were resected and visualized. **(B)** The growth curve of the tumors in each group was analyzed. **(C)** Average tumor volume and weight indicated that CCAT2 inhibited tumor growth *in vivo*. **(D)** Bioluminescent imaging was used to detect SW620 metastasis. **(E)** HE staining was performed to evaluate the EMT potential of SW620 cells in each group (×200). The data are represented as mean ± SD. Three independent biological repeats were used for each analysis. *P < 0.05 **(F)** IHC was performed to evaluate the expression of p-AKT, p-GSK3â, cyclin D1 and cyclin E1.

To further assess the biological roles of CCAT2 in tumorigenesis, we constructed a xenograft nude mouse model (**Figure 8A**). SW620 cells infected with Ad-CCAT2, Ad-shCCAT2 or their controls were subcutaneously injected into the back of nude mice, and tumor volume and weight were measured. CCAT2 promoted the growth of SW620 cells while CCAT2 knockdown inhibited it (**Figures 8B, C**). Bioluminescent imaging was used to detect SW620 metastasis. Remarkably, the metastasis of luciferase-tracking SW620 cells was promoted by CCAT2 overexpression but inhibited by CCAT2 knockdown (**Figure 8D**). Furthermore, HE staining revealed that the overexpression of CCAT2 altered the phenotypes of SW620 cells while CCAT2 knockdown inhibited such alterations (**Figure 8E**). IHC results indicated that CCAT2 promotes the expression of p-AKT, p-GSK3â, cyclin D1 and cyclin E1 while knockdown of CCAT2 inhibited that (**Figure 8F**). These results suggested that CCAT2 promoted the growth and metastasis of CCR cells *in vivo*.

## Discussion

Colorectal cancer (CRC) is the third most common cancer malignancy worldwide. The dysregulation of lncRNAs can promote cell proliferation, resistance to apoptosis, angiogenesis, metastasis, and evasion of tumor suppressors, and is associated with several pathophysiological processes, including cancer, neurodegeneration, as well as autoimmune and cardiovascular diseases (27, 28). The lncRNA CCAT2 is oncogenic in colon cancer and *CCAT2* gene polymorphisms are linked to several types of cancer such as colon, kidney, thyroid, larynx, lung and myeloid cancers in different populations (29–32). CCAT2 has been used as a diagnostic and prognostic biomarker in the treatment of colorectal cancer. In this study, we validated the upregulation of CCAT2 in CRC tissues. Overexpression of CCAT2 remarkably enhanced the proliferation, invasion and wound healing potential of the SW620 and LOVO cells. Previously, *in vivo* research investigated the effect of CCAT2 overexpression in HCT116 cells. Here, we assessed the effects of both overexpression and knockdown of CCAT2 on the growth of SW620 cells. Our results confirmed that CCAT2 promoted the metastasis of SW620 cells *in vivo*, thereby suggesting that it acts as an oncogene in CRC.

To further explore the underlying mechanisms by which CCAT2 acts as an oncogene in CRC, we evaluated its subcellular localization and found that CCAT2 was located mainly in the nucleus. Thus, we hypothesized that CCAT2 may be involved in epigenetic regulation. To test this hypothesis, we performed RNA pull down and mass spectrometry analyses. Our results suggested that CCAT2 might directly bind with TAF15. LncRNA Transient receptor potential cation channel subfamily M member 2-AS (TRMP-AS), an antisense transcript of the *TRMP2* gene, promotes the proliferation of CRC cells by directly enhancing the activity of RNA-binding protein (RBP) TAF 15, which stabilizes *TRMP2* mRNA (33). Using starBase software, we identified TAF15 target genes and found that TAF15 and CCAT2 directly promote the transcriptional activity of *RAB14*.

The majority of Rab proteins promote cancer progression. Rab14 is a member of the RAS oncogene family of small GTPase proteins (34). Rab proteins participate in phagosomal systems and biosynthetic/recycling pathways such as vesicle trafficking, signal transduction and receptor recycling (35). MicroRNA-451 (miR-451) and miR-338-3p act as tumor suppressors in human non-small-cell lung carcinoma (NSCLC) by targeting Ras-related protein 14 (36). Choroideremia-like protein (CHML), a member of the Rab escort protein (REP) family, promotes migration, invasion and metastasis of hepatocellular carcinomas (HCC) cells by facilitating Rab14 recycling (37). GSK3β, a member of the GSK3 family of serine/threonine protein kinases, is aberrantly activated in various cancer types, including colorectal cancer (38–41). Tumor necrosis factor alpha (TNFα), a pro-inflammatory cytokine, induces epithelial-mesenchymal transition (EMT) in human HCT116 cells and thereby promotes CRC invasion and metastasis (37). EMT is a hallmark of the initiation and early growth of primary epithelial cancers (42–45); it gives cells the ability to metastasize and invade tissues, confers them stem cell characteristics, reduces apoptosis and aging, and promotes immunosuppression. However, the mechanistic link between RAB14 and the AKT/GSK-3β signaling remains obscure (46). Here, we found that CCAT2 promoted the growth and metastasis of CRC cells by targeting TAF15 to enhance the transcriptional activation of RAB14, followed by the activation of the AKT/GSK3β signaling pathway.

Our findings elucidated the underlying mechanism by which CCAT2 promotes CRC. LncRNAs act as miRNA/mRNA sponges. The association of lncRNA CCAT2 with miRNA in CRC remains to be elucidated. Nonetheless, our study here showed that CCAT2/TAF15/RAB14/AKT/GSK3β can serve as potential diagnostic and prognostic biomarkers for the treatment of CRC.

## Materials and Methods

### Clinical Samples

A total of 44 paired samples of human CRC and adjacent normal tissues were collected at Shengjing hospital, China Medical University, China. Human colorectal cancer (CRC) tissue and matched adjacent normal tissue from the same patient were collected with the patient consent at the time of operation. The research was approved by the Ethics Committee of Shengjing hospital, China Medical University, China, and was performed in accordance with the Declaration of Helsinki.

### RNA Extraction and Real-Time Quantitative PCR (qPCR)

Total RNAs were isolated from tissues or cells with Trizol reagent (Invitrogen, Shanghai). Five µg of RNA were reverse-transcribed into cDNA using M-MLV reverse transcriptase (Promega, USA). A cDNA template was used to amplify CCAT2. qPCR was carried out using a SYBR Premix Ex Taq kit (TaKaRa, Dalian) in a FAST7500 real-time PCR system (ABI, USA). The specific primer pairs were as follows: CCAT2 forward: 5’CTTCCAGCTCCACCTCTGAC3’, reverse: 5’ GAGCTCAAAGGACGATGAGG3’; RAB14 forward: 5’ GACAGATGCAAGGAATCTCACC 3’, reverse: 5’ GCTTCGAGGAACAATAAGCCAT3’ β-actin forward: 5’ AGTGTGACGTGGACATCCGCAAAG 3’, 5’ ATCCACATCTGCTGGAAGGTGGAC 3’.

### Cell Culture and Transfection

Human CRC cell lines SW620, SW480, HT-29, LOVO, HCT116, DLD-1 and M5 and the normal colorectal epithelial HFC cell line were purchased from the Chinese Academy of Sciences, China. All cell lines were cultured in DMEM medium (Gibco, USA), and supplemented with 10% fetal bovine serum (FBS), 100 IU/ml penicillin, and 100 µg/ml streptomycin. Cells were incubated at 37°C in a humidified chamber supplemented with 5% CO_2_. The adenovirus overexpressing or silencing CCAT2 and their negative controls were established by Genepharma (Shanghai, China). Cells were infected with the adenovirus at a multiplicity of infection (MOI) of 200.

### CCK-8 Assay

After infection for 12 h, SW620 and LOVO cells in each group were seeded onto a 96-well plate and then cultured at 37°C, and 5% CO_2_ for 48 h. Thereafter, CCK-8 assay was carried out at 0, 24, 48 and 72 h. At each time point, 10 μl CCK-8 was added to each well after the medium was replaced. Following incubation for another 4 h at 37°C, absorbance was measured at 450 nm in a microplate reader (Biorad, USA).

### Colony Formation Assay

Following infection for 12 h, the SW620 and LOVO (200 cells per well) cells were transferred to 12-well plates with DMEM medium supplemented with 10% FBS. The cells were cultured at 37°C and 5% CO_2_ for two weeks. Subsequently, the cell colonies were fixed with 4% paraformaldehyde and stained with 0.1% crystal violet (Beyotime, Shanghai, China) for 20 min at room temperature, and the colonies were counted.

### EdU Staining

SW620 and LOVO cells (1.5 × 105 cells/well) were cultured in 24-well plates and infected with Ad-CCAT2, Ad-shCCAT2 or control adenovirus for 24 h. Afterwards, 50 μM EdU (Yuheng, Suzhou, China) was added to these cells and incubated for another 2 h. Afterward, cells were washed thrice with PBS and fixed with 4% paraformaldehyde, followed by staining with Apollo staining solution and DAPI. Finally, cell proliferation was assessed by counting cells using a light microscope (Nikon, Japan).

### Transwell Cell Migration Assay

12 h after of infection, SW620 and LOVO cells were suspended in serum-free DMEM medium. Cells at a density of 3×105 cells/ml were seeded onto the top chamber of Transwell 24-well plates (Corning, USA). Then, 600 μl DMEM medium containing 15% FBS was added into the lower chamber. After being cultured at 37°C for 24 h, the cells under the surface of the lower chamber were fixed with 4% paraformaldehyde followed by staining with crystal violet (0.1%, Beyotime, Shanghai, China) at room temperature for 30 min. Cell migration was evaluated by counting the cells that had migrated into the filters using an optical microscope (Nikon, Japan).

### Transwell Cell Invasion Assay

In the invasion assay, 50 μl BD Matrigel™ (BD, USA) was added on to the transwell upper chamber and placed in a 37°C incubator for 2 h to solidify. The following experiment was performed similarly as above mentioned in the migration assay.

### Wound Healing Assay

Wound-healing assay was used to measure cell migration capacity in response to CCAT2 treatment. SW620 and LOVO cells were seeded in 6-well plates after infection until the confluence reached 90%. The cell monolayers were scratched with a micropipette tip to make a gap. The cell culture surface was washed three times with PBS to remove cellular debris and incubated in DMEM medium containing 2% FBS. Images were taken with an optical microscope (Nikon, Japan) 48 h later and the distance between two lines was measured and quantitated.

### RNA Immunoprecipitation (RIP) and RNA Pull Down Assay

RIP and RNA pull-down assay were carried out using EZ-Magna RIP RNA-Binding Protein Immunoprecipitation Kit (Millipore, USA) and Pierce Magnetic RNA–Protein Pull-Down Kit (Thermo, USA), respectively, according to the manufacturers’ instructions. The CCAT2 probe labeled with biotin was commercially purchased from GenePharma (Shanghai, China) and then incubated with streptavidin magnetic beads (Invitrogen, USA) for 1.5 h at 37°C, followed by incubation with the lysates from SW620 and LOVO cells at 4°C overnight. Finally, total extracts of SW620 and LOVO cells were prepared and was subjected to silver staining and Western blotting.

### Western Blot Analysis

Protein samples from cells were extracted by RIPA buffer (Beyotime, China) and separated in 10% SDS-PAGE gels and then transferred to PVDF membranes (Millipore, USA). The membranes were incubated with primary antibodies (anti-p-AKT-phosphoT308, 1:1000, abcam, England; anti-AKT, 1:1000, abcam, England; anti-p-GSK3β-phosphoS9, 1:1000, abcam, England; anti-GSK3β, 1:1000, abcam, England; cyclinD1, 1:500, Proteintech, China; cyclinE1, 1:500, Proteintech, China; p21, 1:1000, Proteintech, China; β-catenin, 1:500, Proteintech, China; vimentin, 1:500, Proteintech, China; E-cadherin, 1:500, Proteintech, China; N-cadherin, 1:500, Proteintech, China; TAF15, 1:500, Proteintech, China; RAB14, 1:500, abcam, England) overnight and then incubated with the corresponding secondary antibody. Band intensity was measured using chemiluminescence (ECL) system kit according to the manufacturer’s instructions (Solarbio, Beijing, China). The optical densities (OD) value was analyzed with ImageJ software (NIH, Bethesda, MD, USA).

### FISH Assay

For Fluorescence *in situ* hybridization (FISH) assay, 48 h after adenovirus infection, cells were fixed with 4% paraformaldehyde for 20 min, permeabilized with 0.05% triton for 5 min, and then blocked with10% donkey serum for 1 h. After fixation, cells were incubated with CCAT2 probe (GenePharma, China) for 1 h. The nuclei were stained with DAPI for 5 min, and the optical microscope (Nikon, Japan) was used for observation. The sequence of the probe is Biotin-TTTTCCATTTGTCGAGAGAGCGGGTGTTCTCTGAGGACCGCACACG.

### Dual Luciferase Reporter Assay

Based on bioinformatics predictions, we found that RAB14 was regulated by TAF15. The promoter segments of RAB14 were obtained by PCR and inserted into pGL4.10 vector. 250 ng reporter vector, 250 ng overexpressing vector or siRNA, and 10 ng pRL-SV40 vector were transfected with Lipofectamine 3000 (Invitrogen, USA) according to the instructions. After 48 h, cells were lysed in 100 μl of passive lysis buffer. The firefly luciferase activity and the Renilla activity were determined using a Dual-Luciferase^®^ Reporter Assay System. For each experiment, firefly luciferase activity was normalized to Renilla activity.

### Analysis of Tumor Xenografts

All the animal experiments were approved by the Ethic Committee of Shengjing hospital, China Medical University, China. For the *in vivo* growth assay, SW620 cells were injected subcutaneously into 6-week-old female BALB/C nude mice (1×10^7^ cells/mice in 100 μl of DMEM). After 7 d, sh-NC, CCAT2, shNC and shCCAT2 adenovirus were injected into the tumor body. Meanwhile, the length and width of tumors of all mice were measured every five days. Tumor volume was determined according to the equation: V = (L ×W2)/2, where V is the volume, L is the length and W is the width of the tumor. On the 6th week of injection, mice were killed and tumors were harvested, weighed and imaged.

### Analysis of Tumor Metastasis

We prepared SW620 cells as follows after adenovirus infection: six week old female nude mice were anaesthetized using 1% pentobarbital (40mg/kg); then, cells were transplanted intrasplenically into the mice. At the endpoint, mice were again anaesthetized using 1% pentobarbital (160mg/kg) and observed in the IVIS Spectrum living image system (PerkinElmer, USA). The images were obtained and analyzed using ImageJ software (version 1.42q).

### Hematoxylin Eosin (HE) Staining

Sliver-stained samples were separated and placed in 10% formalin overnight and embedded in paraffin. Then, the tissues were sliced into 5 μm-thick sections and fixed on a glass slide. The staining procedures were performed according to the manufacturer’s instructions (Solarbio, Beijing, China). Briefly, the sections were soaked in xylene, ethanol in gradient concentration, and hematoxylin, respectively, and sealed with resin. Finally, the morphology was observed and observed under a light microscope.

### Statistical Analysis

Statistical analyses were carried out using SPSS version 20.0 software and Graph Pad Prism version 6.0. The data are presented as mean ± standard deviation (SD). A Student’s *t*−test was used to evaluate the significance of the difference between two groups. P<0.05 was considered to represent a statistically-significant difference.

## Data Availability Statement

The original contributions presented in the study are included in the article/supplementary material. Further inquiries can be directed to the corresponding author.

## Ethics Statement

Written informed consent was obtained from the individual(s) for the publication of any potentially identifiable images or data included in this article.

## Author Contributions

DW and ZL did the experiments and analyzed the data. HY supervised the research. All authors contributed to the article and approved the submitted version.

## Conflict of Interest

The authors declare that the research was conducted in the absence of any commercial or financial relationships that could be construed as a potential conflict of interest.

## Publisher’s Note

All claims expressed in this article are solely those of the authors and do not necessarily represent those of their affiliated organizations, or those of the publisher, the editors and the reviewers. Any product that may be evaluated in this article, or claim that may be made by its manufacturer, is not guaranteed or endorsed by the publisher.

## Abbreviations

CRC, Colorectal cancer; lncRNAs, long noncoding RNAs; CCAT2, colon cancer-associated transcript 2; IF, immunofluorescence; EMT, epithelial-mesenchymal transition; RIP, RNA immunoprecipitation.
